# Studying the association complex formation of atomoxetine and fluvoxamine with eosin Y and its application in their fluorimetric determination

**DOI:** 10.1098/rsos.170943

**Published:** 2018-03-14

**Authors:** Sayed M. Derayea, Mahmoud A. Omar, Ahmed A. Abu-hassan

**Affiliations:** 1 Department of Analytical Chemistry, Faculty of Pharmacy, Minia University, Minia, Egypt; 2 Department of Pharmaceutical Analytical Chemistry, Faculty of Pharmacy, Al-Azhar University, Assiut branch, Assiut, Egypt

**Keywords:** atomoxetine, fluvoxamine, eosin Y, association complex, spectrofluorometry, pharmaceutical analysis

## Abstract

A simple, sensitive and non-extractive spectrofluorimetric method has been developed and validated for the determination of two psychoanaleptic drugs, atomoxetine and fluvoxamine, in pure forms and pharmaceutical dosage forms. The proposed method is based on the formation of binary complexes between eosin Y and the studied drugs in the presence of a Teorell–Stenhagen buffer. The quenching of the native fluorescence of eosin Y due to complex formation with the studied drugs was measured spectrofluorimetrically at 545 nm after excitation at 302 nm. At the optimum reaction conditions, the fluorescence quenching values (Δ*F*) and concentrations were rectilinear over the concentration ranges of 0.2–2.2 and 0.3–2.2 µg ml^−1^ for atomoxetine and fluvoxamine, respectively. The developed method was successfully applied for the determination of the studied drugs in their pharmaceutical formulations with average percentage recoveries of 100.13 ± 0.66 and 99.69 ± 0.44 for atomoxetine and fluvoxamine, respectively (*n* = 5), without interference from common excipients.

## 1. Introduction

**Figure 1.**
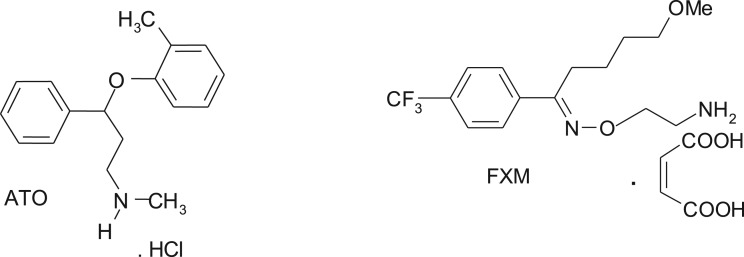
Chemical structures of atomoxetine hydrochloride (ATO) and fluvoxamine maleate (FXM).

Atomoxetine (ATO) and fluvoxamine (FXM) are psychoanaleptic drugs used for stimulating the mood and correcting depressive conditions. Atomoxetine hydrochloride (figure 1) is a selective inhibitor of the presynaptic reuptake of norepinephrine. It has been approved by the FDA as the first non-stimulant drug for the treatment of attention deficit/hyperactivity disorder (ADHD). ADHD is the most common neurobehavioural disorder of childhood and the symptoms of some patients persist to adulthood. It has been marked by symptoms of inattention, hyperactivity and impulsiveness that impair academic and social functioning [1,2].

Fluvoxamine maleate (figure 1) is a second-generation antidepressant used for the treatment of a variety of depressed states [3]. The selective mode of action produces a different side-effect profile to that demonstrated by the tricyclic antidepressants [4].

Literature survey reveals that there are several methods for the determination of the investigated drugs. These methods include spectrophotometry [5–10], spectrofluorimetry [11–13], liquid chromatography (HPLC) [14–17], gas chromatography (GC) [18,19], and electrochemical [20,21] and capillary electrophoretic methods [22,23].

Liquid chromatography requires expensive instruments and consumes large volumes of highly pure solvents. In addition, tedious and time-consuming sample pre-treatment steps are needed. Gas chromatography, electrochemical and electrophoretic methods used sophisticated apparatus and require well-experienced personnel. Although spectrophotometric methods are simple techniques, they have a limited sensitivity and narrow concentration range. On the other hand, spectrofluorimetric methods are more selective, highly sensitive and require a simple apparatus and apply very simple sample preparation. However, the reported spectrofluorimetric methods for the cited drugs were carried out using expensive reagents [11] or hazardous organic solvents at elevated temperatures for long time [12,13].

Eosin Y, tetrabromofluorescein disodium salt, is a yellowish-red dye with green fluorescence. It is an acidic dye that possesses a single carboxyl group. Eosin Y has been applied for the determination of several basic drugs through the formation of binary [24,25] or ternary [26,27] complexes.

In the present work, the ion-pairs between the investigated drugs and eosin Y were employed for the development of a simple and sensitive spectrofluorimetric method for the studied drugs. After full optimization and validation, the method was applied for the determination of the studied drugs in their pharmaceutical formulations.

## 2. Material and methods

### 2.1. Apparatus

All spectrofluorimetric measurements were carried out on a SCINCO FluoroMate (FS-2, Korea). The spectrometer was equipped with a 150 W Xe-arc lamp, a PMT (photo multiplier tube) detector for excitation and emission and 1 cm matched quartz cells. The slit width for both excitation and emission monochromators was set at 10 nm.

A pH-meter, model AD11P (Adwa, Romania), super-mixer (GEMMY industrial CORD, Taiwan, R.O.C.) and bath sonicator (SONICOR SC-101TH) were also used. An electronic single pan balance (Precisa XB 220A, Switzerland) was used for weighing. Distilled water was prepared by a water distiller (TYUMEN-MIDI-A0-25 MO, Russia).

### 2.2. Materials and reagents

ATO was obtained as a gift from MASH Premiere for Pharmaceutical Industries (Badr City, Cairo, Egypt) and FXM was obtained as a gift from Pharaonia Pharmaceuticals (Alexandria, Egypt). Atomox apex capsules (Multi-Apex Pharma S.A.E, Badr City, Cairo, Egypt) contained 10 mg ATO per capsule. Faverin® tablets (Abbott Healthcare SAS) contained 50 mg FXM per tablet.

Eosin Y (Merck, Darmstadt, Germany) was prepared as 0.5 mM in distilled water. Teorell–Stenhagen buffer solution [28] of pH 2.0–4.0 was prepared by mixing the appropriate volumes of 1 M phosphoric acid, 1 M citric acid, 1 M boric acid and 1 M sodium hydroxide. These solutions were mixed together in different proportions to give the required pH, keeping the total buffer strength of 1 M concentration.

### 2.3. Preparation of standard solutions

The standard stock solution of ATO and of FXM was prepared in a 100 ml volumetric flask by dissolving 10 mg of the drug in distilled water. The stock solution was further diluted with distilled water to obtain working standard solutions in the concentration range of 2–22 µg ml^−1^.

### 2.4. General analytical procedure

Into a series of 10 ml volumetric flasks, 1.0 ml of the drug solution (3–22 µg ml^−1^ and 2–22 µg ml^−1^ for FXM and ATO, respectively) was transferred. Then 1 ml of eosin Y (0.5 mM) for FXM and 1.4 ml for ATO was added followed by 1 ml of 1 M Teorell–Stenhagen buffer (pH 3.0 for ATO and 3.2 for FXM). The volume was completed to the mark with distilled water and the fluorescence intensity was measured at 545 nm after excitation at 302 nm. A blank experiment was carried out simultaneously, in the same manner, omitting the drug solution. The difference in the fluorescence intensity in the absence and presence of the drug (Δ*F*) was plotted versus the final drug concentration.

### 2.5. Analysis of dosage forms

#### 2.5.1. Faverin® tablets

An accurately weighed amount of 10 finely powdered tablets equivalent to 50 mg of FXM was transferred to a 100 ml volumetric flask containing 30 ml distilled water. The mixture was sonicated for about 15 min and then completed to the mark with distilled water. The solution was filtered and the first portion of the filtrate was discarded. A portion of the filtrate was diluted quantitatively with the same solvent to give final concentration within the working range. The drug content of the final solution was analysed with the general recommended procedure.

#### 2.5.2. Atomox apex capsules

Contents of 20 capsules were evacuated and weighed. An amount equivalent to 50 mg of ATO was transferred to a 100 ml volumetric flask containing 20 ml distilled water. The solution was sonicated for 15 min and then completed to the mark with distilled water. The solution was filtered and a portion of the filtrate was further diluted and treated as in the general recommended procedure.

## 3. Results and discussion

**Figure 2.**
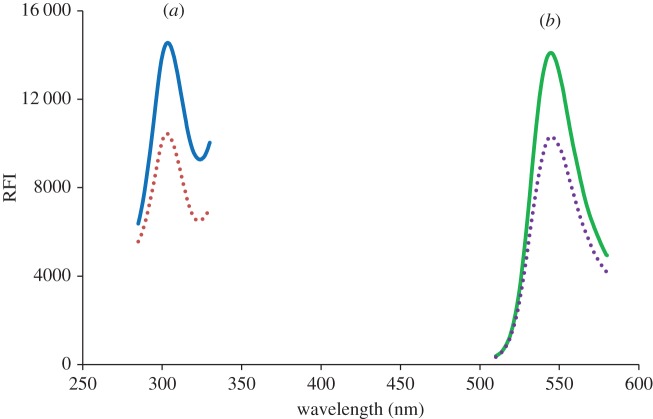
Excitation (*a*) and emission (*b*) spectra of eosin Y (5 × 10^−5^ and 7 × 10^−5^ M) (dotted lines) and its reaction product with 2 µg ml^−1^ atomoxetine (solid lines). RFI, relative fluorescence intensity.

The aqueous solution of eosin showed a native fluorescence activity measured at 545 nm after excitation at 302 nm. The addition of the studied drug solution to the reagent solution significantly decreased the fluorescence intensity (figure 2). The observed fluorescence quenching effect is due to the formation of an ion pair complex between the studied drugs and eosin Y. The magnitude of the decrease of fluorescence intensity of the dye (Δ*F*) was directly proportional to the concentration of the drugs.

### 3.1. Optimization of experimental conditions

Different experimental parameters affecting the development and stability of the reaction product were carefully studied and optimized. Such factors were changed individually while the others were kept constant. These factors included pH, type and concentration of the buffer, concentration of eosin and diluting solvents.

#### 3.1.1. Effect of buffer concentration and pH

**Figure 3.**
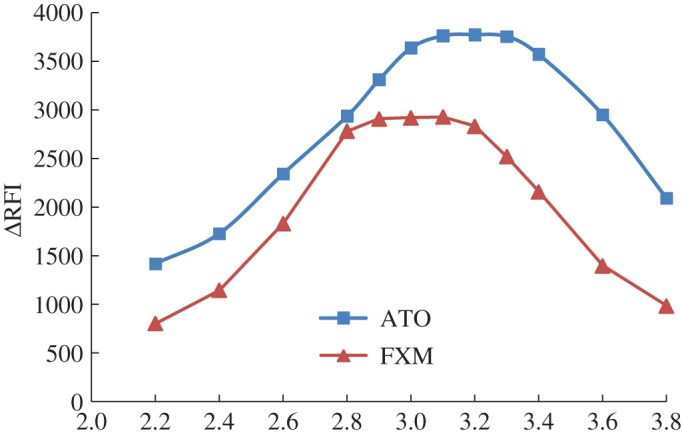
Effect of pH of Teorell–Stenhagen buffer on the fluorescence quenching of eosin Y using 2 µg ml^−1^ of the investigated drugs.

**Figure 4.**
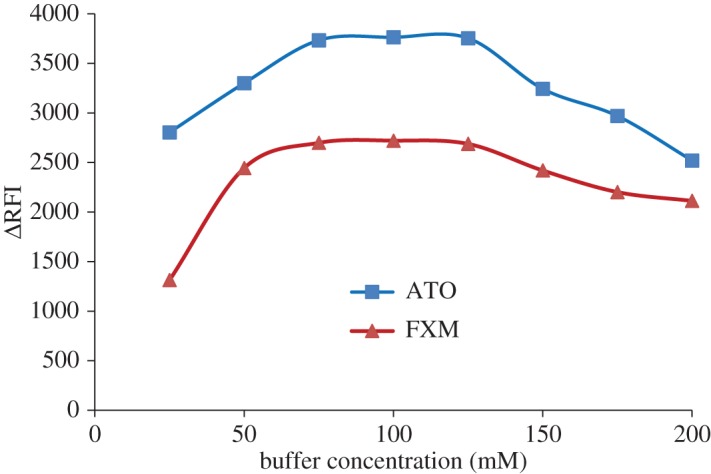
Effect of buffer concentration on the decrease in the fluorescence intensity of eosin using 2 µg ml^−1^ of the investigated drugs.

The influence of pH was studied using a Teorell–Stenhagen buffer in the pH range 2.0–4.0. It was found that the maximum Δ*F* was attained at pH 3.2 ± 0.2 and 3.0 ± 0.2 for ATO and FXM, respectively. Higher or lower pH values reduced the obtained results as shown in figure 3. The effect of buffer concentration on the quenching of the fluorescence intensity of eosin Y was studied over the range 25–200 mM of a Teorell–Stenhagen buffer solution. It was found that 100 mM was sufficient to produce the maximum Δ*F*, as shown in figure 4.

#### 3.1.2. Effect of type of buffer

Different types of buffer were tested to study their effect on the fluorescence quenching of eosin Y at the optimum pH. Four types of buffer solutions including Teorell–Stenhagen, acetate buffer, Mcllvaine buffer and Britton Robinson buffers were studied. Maximum fluorescence quenching (Δ*F*) was obtained when a Teorell–Stenhagen buffer was used.

#### 3.1.3. Effect of concentration of eosin

**Figure 5.**
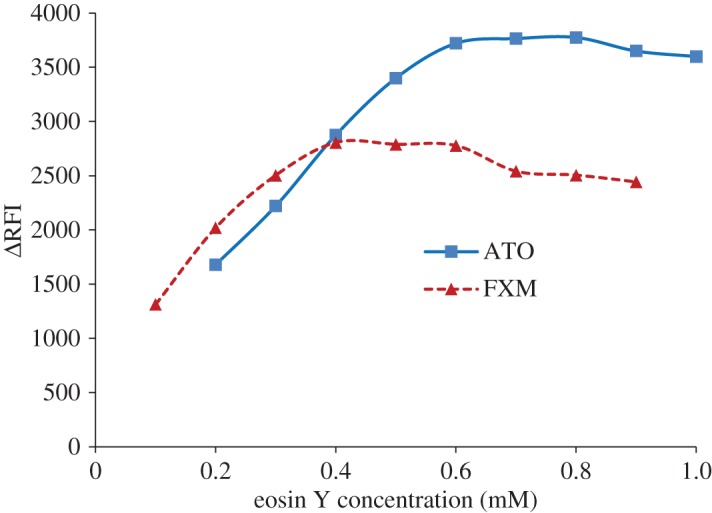
Effect of the eosin Y concentration on the ion-pair complexation with the studied drugs (2 µg ml^−1^).

Increasing the concentration of eosin Y resulted in an increase in the fluorescence quenching (Δ*F*). Maximum values were obtained when using 0.5 ± 0.1 and 0.7 ± 0.1 mM of eosin Y reagent for FXM and ATO, respectively. Higher concentrations of the reagent slightly decreased the obtained results, as shown in figure 5.

#### 3.1.4. Effect of diluting solvents

The formed complex was diluted with different solvents such as water, methanol, ethanol and acetonitrile. Water was found to be the most appropriate solvent for dilution as other solvents gave lower results. Water is readily available, cheap and the best green solvent. Therefore, the proposed method is advantageous over many of the reported methods which use organic solvents such as chloroform [13] and acetonitrile [12].

### 3.2. The stoichiometry of the reaction

**Figure 6.**
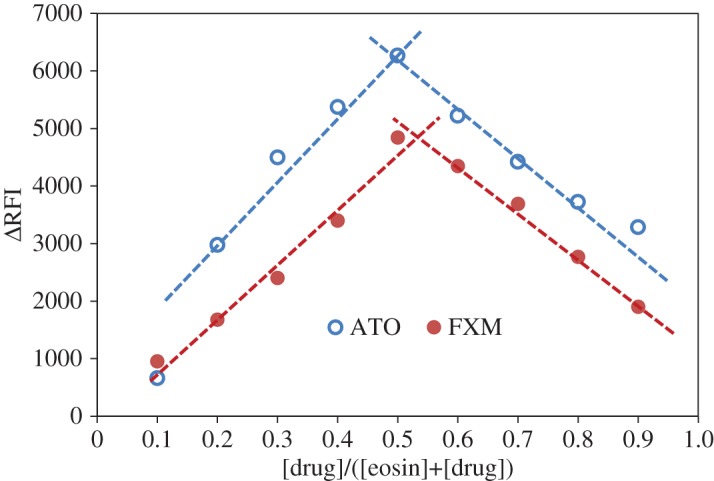
Jobs plots for molar ratio determination using equimolar solutions (0.5 mM) of both eosin Y and the investigated drugs at optimum pH.

Job's method of continuous variation [29] was used to study the molar ratios of the formed ion-pair complexes. Equimolar solutions (5 × 10^−4^ M) of both the drug and the reagent were prepared and mixed in different molar ratios keeping total molar concentration constant. The results of Job's method revealed that the ratio of eosin Y to the drug was 1 : 1 for both ATO and FXM, as shown in figure 6.

**Figure 7.**
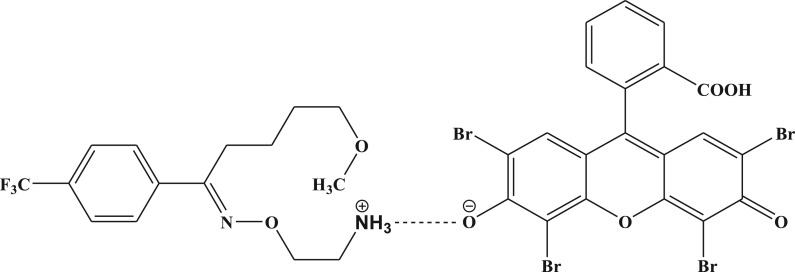
The proposed structure of the formed ion-pair association between fluvoxamine (as an example) and eosin Y at pH 3.0 in aqueous solution.

This ratio is in agreement with the presence of only one basic nitrogen atom in both drugs. At the specified pH (3.0–3.2), the nitrogen atom would be protonated to form a drug cation. At the same time, the hydroxyl group of eosin Y will dissociate to produce an eosinate anion which will interact with the drug cation to form the ion-pair complex through electrostatic forces. A proposal of the possible structure of the ion-pair association between FXM and eosin Y is illustrated in figure 7.

### 3.3. Method validation

After optimization of reaction conditions, the proposed method was fully validated according to ICH guidelines [30]. The validation parameters included linearity range, accuracy, precision, limit of detection, limit of quantitation and robustness. All results were expressed as percentages, with representation of the number of determinations for each value.

#### 3.3.1. Linearity range

The general analytical procedures were applied on a series of standard solutions of the studied drugs having different concentrations, and the fluorescence intensities of the solutions were measured. The calibration curves for the studied drugs were constructed by plotting difference in the relative fluorescence intensity (ΔRFI) versus the final drug concentrations. Linear regression analysis of the data was carried out and the analytical parameters were calculated. The parameters of the proposed method are presented in table 1. The fluorescence quenching values (Δ*F*) were rectilinear with the drug concentrations in the ranges of 0.2–2.2 and 0.3–2.2 µg ml^−1^ for ATO and FXM, respectively, with a correlation coefficient of 0.9998.

**Table 1.** Regression equation and validation parameters for the proposed spectrofluorimetric method. LOD is limit of detection and LOQ is limit of quantitation.

parameters	atomoxetine	fluvoxamine
linear range (μg ml^−1^)	0.2–2.2	0.3–2.2
intercept (a)	1057.2	421.5
slope (b)	1362.2	1170.7
correlation coefficient (*r*)	0.9998	0.9998
LOQ µg ml^−1^	0.19	0.26
LOD µg ml^−1^	0.06	0.08

#### 3.3.2. Accuracy

The accuracy of the method was checked by applying the general analytical procedure on five concentrations within the specified range. Three replicate measurements of each concentration level were recorded. The results were presented as percentage recovery ± standard deviation. The results in table 2 show the close agreement between the measured and true values.

**Table 2.** Evaluation of accuracy of the analytical procedure of the studied drugs.

Drug	% recovery^a^ ± s.d.				
conc. (μg ml^−1^)	0.4	0.8	1.0	1.4	2.0
atomoxetine	101.39 ± 0.92	100.89 ± 1.01	99.43 ± 0.74	98.76 ± 0.55	99.29 ± 0.30
fluvoxamine	98.69 ± 1.73	99.42 ± 1.04	100.04 ± 0.55	99.42 ± 0.40	100.70 ± 0.37

^a^Average of three determinations, s.d., standard deviation.

#### 3.3.3. Precision

Precision was estimated at two levels, repeatability and intermediate precision. The repeatability (intra-day precision) was performed by repeating the analysis three successive times within the same day, and the intermediate (inter-day) precision was performed on three successive days. The low values of the standard deviations (less than 2.0) indicate the high precision of the proposed methods (table 3).

**Table 3.** Evaluation of intra-day and inter-day precision of the proposed method.

	% recovery ± s.d.	
drug concentration (μg ml^−1^)	atomoxetine	fluvoxamine
intra-day precision^a^		
0.5	100.74 ± 0.90	99.34 ± 1.04
1.0	98.85 ± 1.07	99.07 ± 0.69
1.5	100.60 ± 0.54	98.94 ± 1.07
inter-day precision^a^		
0.5	101.18 ± 1.62	100.37 ± 1.80
1.0	100.12 ± 0.58	99.16 ± 0.79
1.5	98.98 ± 1.24	101.00 ± 0.71

^a^Mean value of three determinations, s.d., standard deviation.

#### 3.3.4. Limits of detection and quantification

The sensitivity of the proposed method was evaluated by calculating detection and quantitation limits. These limits were calculated using the formula: LOD = 3.3*σ*/*S* and LOQ = 10*σ*/*S* for limits of detection and quantitation, respectively, where *σ* is the standard deviation of intercept and *S* is the slope of the calibration curve. The obtained LOD values were 0.06 and 0.08 µg ml^−1^, while LOQ values were 0.19 and 0.26 µg ml^−1^ for ATO and FXM, respectively (table 1).

#### 3.3.5. Robustness

The robustness of the procedure was assessed by evaluating the influence of small variation in experimental variables (pH, buffer concentration and reagent concentration) on the analytical performance of the method. From the obtained results shown in table 4, it was found that the small variations in any of the studied variables did not significantly affect the results of the method as the obtained s.d. did not exceed 2%. This is an indication of the reliability of the proposed method during routine work.

**Table 4.** Robustness of the proposed method for the determination of the studied drugs (1.2 µg ml^−1^).

		% recovery^a^ ± s.d.	
parameters	value	atomoxetine	fluvoxamine
pH	−0.1	99.89 ± 0.61	99.34 ± 1.05
	+0.1	101.07 ± 0.53	99.67 ± 0.74
eosin concentration	−0.1 mM	99.72 ± 0.94	98.79 ± 1.22
	+0.1 mM	101.56 ± 0.39	100.59 ± 1.04
buffer concentration	−20 mM	98.87 ± 0.43	99.52 ± 0.93
	+20 mM	99.17 ± 1.11	98.55 ± 1.03

^a^Mean value of three determinations, s.d. is the standard deviation.

### 3.4. Application to pharmaceutical formulations

The general analytical procedure was applied to determine the drug content of different commercial dosage forms. The obtained results were statistically compared with those of the reported methods [9,10] with respect to accuracy and precision (table 5). No significant difference was found between the results of both methods as calculated *t*- and *F*-test values did not exceed the tabulated values. The obtained excellent recoveries prove that there is no interference from the frequently encountered excipients. This indicates the suitability of the proposed method for the determination of the studied drugs in their dosage forms in quality control laboratories.

**Table 5.** Analysis of atomoxetine and fluvoxamine in pharmaceutical formulations using the proposed and reported methods.

			% recovery^a^ ± s.d.	
dosage forms	labelled claim (mg)	found mg	proposed method	reported method
Atomox apex capsules	10	10.013	100.13 ± 0.66 (*t* = 0.81, *F* = 3.20)^b^	100.63 ± 1.19^10^
Faverin® tablets	50	49.845	99.69 ± 0.44 (*t* = 1.27, *F* = 2.60)^b^	99.22 ± 0.71^9^

^a^The value is the average of five determinations for both the proposed and reported methods.

^b^Tabulated values at 95% confidence limit are *t* = 2.306, *F* = 6.338.

Advantages of the proposed method over the previously published spectrofluorimetric methods [11–13] include the use of a widely available and cheaper reagent (eosin Y) compared with fluorescamine, which was reported for the determination of FXM [11]. Additionally, the current method is very rapid and the reaction between the studied drugs and eosin was instantaneous at room temperature. Therefore, there was no need for standing time before the measurements. The reported methods [12,13] using 4-chloro-7-nitro-2,1,3-benzoxadiazole as a derivatizing agent for the determination of ATO and FXM required heating the reaction mixture for 20 min at an elevated temperature. Moreover, the method is environmentally safer than other reported methods, which were performed in hazardous organic solvents such as acetonitrile [12] and chloroform [13].

## 4. Conclusion

A sensitive, economic and validated spectrofluorimetric method was developed for the determination of ATO and FXM in pharmaceutical preparations, depending on the reaction of their amino groups with eosin Y. The most important advantage of the method is that the ion-pair formed is measured directly without the need for pretreatment of the sample or extraction with organic solvent. Hence, it can be applied to the quality control of the studied drugs in their dosage forms.

## Data accessibility

This article does not contain any additional data.
